# Supplementary material for: Modelling Skylarks (Alauda arvensis) to Predict Impacts of Changes in Land Management and Policy: Development and Testing of an Agent-Based Model
Source: PLoS One. 2013 Jun 6;8(6):e65803. doi: 10.1371/journal.pone.0065803 (PMC3675089; doi:10.1371/journal.pone.0065803)
Supplement: Supporting Information S4 — The skylark ODdox as a zipped archive. (ZIP) [file pone.0065803.s004.zip › Skylark_ODdox/functions_func_0x6f.html]

ALMaSS Skylark ODdox: Class Members - Functions


ALMaSS Skylark ODdox
 2.0


- Main Page
- Related Pages
- Classes
- Files

- Class List
- Class Index
- Class Hierarchy
- Class Members

- All
- Functions
- Variables
- Typedefs

- a
- b
- c
- d
- e
- f
- g
- h
- i
- j
- k
- l
- m
- n
- o
- p
- r
- s
- t
- u
- v
- w
- y
- z
- ~

### - o -

- Occupy()
  : SkTerritories
- OldDays()
  : Calendar
- On\_FoodSupply()
  : Skylark\_Nestling
  , Skylark\_Base
- OnAddNestling()
  : Skylark\_Male
- OnAddPreFledgeling()
  : Skylark\_Male
- OnArrayBoundsError()
  : TALMaSSObject
- OnBreedingSuccess()
  : Skylark\_Female
- OnBreedSuccess()
  : Skylark\_Female
- OnBroodDeath()
  : Skylark\_Female
  , Skylark\_Male
- OnBroodDesertion()
  : Skylark\_Male
- OnClutchDeath()
  : Skylark\_Female
- OnDadDead()
  : Skylark\_Nestling
- OnDeserted()
  : Skylark\_Nestling
- OnEggHatch()
  : Skylark\_Male
- OnEggsHatch()
  : Skylark\_Female
- OnEvicted()
  : Skylark\_Male
- OnFarmEvent()
  : TAnimal
  , Skylark\_Clutch
  , Skylark\_Nestling
  , Skylark\_PreFledgeling
  , Skylark\_Female
  , Skylark\_Male
- OnFoodMessage()
  : Skylark\_Male
- OnMaleNeverComesBack()
  : Skylark\_Female
- OnMateDying()
  : Skylark\_Female
  , Skylark\_Male
- OnMateHomeless()
  : Skylark\_Female
- OnMateLeaving()
  : Skylark\_Male
- OnMateNeverComesBack()
  : Skylark\_Male
- OnMumGone()
  : Skylark\_Clutch
- OnNestlingDeath()
  : Skylark\_Male
- OnNestLocation()
  : Skylark\_Male
- OnNestPredatation()
  : Skylark\_Male
- OnPairing()
  : Skylark\_Male
- OnPreFledgelingDeath()
  : Skylark\_Male
- OnPreFledgelingMature()
  : Skylark\_Male
- OnReHouse()
  : Skylark\_Male
- OnSetMyClutch()
  : Skylark\_Female
- OnStopFeedingChicks()
  : Skylark\_Female
- OnYouHaveBeenEaten()
  : Skylark\_Nestling
- OpenFile()
  : probe\_data
- OpenForAppendToFile()
  : probe\_data
- OpenTheBreedingPairsProbe()
  : Population\_Manager
  , Skylark\_Population\_Manager
- OpenTheBreedingSuccessProbe()
  : Population\_Manager
  , Skylark\_Population\_Manager
- OpenTheCIPEGridOutputProbe()
  : Population\_Manager
- OpenTheFledgelingProbe()
  : Skylark\_Population\_Manager
  , Population\_Manager
- OpenTheMonthlyRipleysOutputProbe()
  : Population\_Manager
- OpenTheReallyBigProbe()
  : Population\_Manager
- OpenTheRipleysOutputProbe()
  : Population\_Manager
- operator()()
  : CompareStateR
  , CompareState
  , CompareFarmNum
  , CompareX
  , CompareY
- operator<()
  : LowPriPair< ELEMTYPE >
- OptimiseHabitatSearchingOrder()
  : Skylark\_Male
- Orchard()
  : Orchard
- OrchardBand()
  : OrchardBand
- OrchardBorderAdd()
  : Landscape
- OrchardGrass()
  : OrchardGrass
- OrganicCattle()
  : OrganicCattle
- OrganicPig()
  : OrganicPig
- OrganicPlant()
  : OrganicPlant


- Generated on Thu Jan 10 2013 13:15:39 for ALMaSS Skylark ODdox by
   1.8.1.1
